# Evaluation of the Clinical and Radiological Outcomes of Pulpotomized Primary Molars Treated with Three Different Materials: Mineral Trioxide Aggregate, Biodentine, and Pulpotec. An In-vivo Study

**DOI:** 10.7759/cureus.4803

**Published:** 2019-06-02

**Authors:** Raparla Mythraiye, VV Rao, MS Minor Babu, Martha Satyam, Punithavathy R., Chandrika Paravada

**Affiliations:** 1 Pedodontics and Preventive Dentistry, Lenora Institute of Dental Sciences, Rajahmundry, IND; 2 Pedodontics and Preventive Dentistry, Lenora Institute of Dental Sciences, Rajahmundry , IND

**Keywords:** pulpotomy, mta, biodentine, pulpotec, formocresol, pulptherapy

## Abstract

Introduction

The main treatment objective of pediatric dentistry lies in maintaining the integrity of the arches. The loss of primary teeth at an early age causes malocclusion. Primary teeth are considered the best space maintainers in the arch. Hence, every effort should be directed to preserve these teeth as far as possible. One of the most important goals of pediatric dentistry is the restoration of carious primary teeth and the maintenance of optimal oral health.

Aim

The aim of this study is to compare and evaluate different pulpotomy materials like mineral trioxide aggregate (MTA), Biodentine, and Pulpotec in primary molars.

Materials and methods

In the present study, 84 primary molars were selected for the pulpotomy procedure and randomly assigned to one of the three groups of MTA, Biodentine, and Pulpotec, allocating 28 primary molars to each group. The pulpotomy procedure was performed on all selected teeth and followed by permanent restoration with stainless steel crowns. All the molars were evaluated, both clinically and radiographically, at an interval of one, three, and six months.

Results

At the end of the first month, there were no adverse clinical and radiographical findings observed in all three groups. At the end of the third month, Group I showed 96% clinical and radiographical success, Group II showed 100% clinical and 96% radiographical success, and Group III showed 100% clinical and radiographical success. At the end of the sixth month, Group I showed 96% clinical and radiographical success, Group II showed 100% clinical and 90% radiographical success and Group III showed 100% clinical and radiographical success. The observations were subjected to statistical analysis using Fisher’s exact test and the Chi-square test.

Conclusion

MTA, Biodentine, and Pulpotec can be used as materials of choice for pulpotomy. Furthermore, Pulpotec appeared to be clinically and radiographically more successful than MTA and Biodentine.

## Introduction

The goal of pediatric dentistry is maintaining arch integrity by preserving optimal oral health. Pulpotomy and pulpectomy are the two procedures for treating infected pulp tissue in order to prevent dental abscesses and loss of teeth.

Pulpotomy is conservative treatment performed to remove the inflamed coronal portion of the pulp by preserving the vitality of the remaining radicular pulp. Formocresol has been a popular pulpotomy medicament in the primary dentition and is still the most universally used pulp medicament for primary teeth. The use of formocresol in humans raised several concerns, and alternatives have been proposed. Mineral trioxide aggregate (MTA), Biodentine, and Pulpotec are recently introduced materials that have been considered as alternatives to formocresol for pulpotomies in primary teeth with exposed pulps. The present study was aimed at evaluating and comparing the success of pulpotomy using MTA, Biodentine, and Pulpotec clinically and radiographically.

## Materials and methods

The present study was conducted in the Department of Pedodontics and Preventive Dentistry, Lenora Institute of Dental Sciences, Andhra Pradesh. Healthy children in the age group of five to nine years, with no history of systemic illness having restorable primary first and second molars, with deep carious lesions, were included in the study. Eighty-four primary molars were selected for the pulpotomy procedure and randomly assigned to one of the three groups, viz. MTA, Biodentine, and Pulpotec, allocating 28 primary molars to each group.

For each child, access preparation was done under local anesthesia and isolation. Following pulpal exposure, the superficial pulp was removed with a sterile, sharp spoon excavator. Samples were assigned randomly to one of the three groups. After achieving hemostasis, in Group I, MTA paste; in Group II, Biodentin; and in Group III, Pulpotec paste was placed in the pulp chamber and condensed lightly with a moistened cotton pellet. The teeth of the children in all three groups were filled with a thick layer of Zinc Oxide Eugenol, followed by Glass Ionomer Cement (GIC) type IX and a stainless steel crown within 10 days.

All cases were evaluated both clinically and radiographically after a period of one month, three months, and six months. Clinical success was defined as the absence of spontaneous pain, acute abscess, fistula, or excessive mobility. Radiographic success was defined as the presence of a normal periodontal ligament space, the absence of furcal radiolucency, pathologic root resorption, or root canal calcification. All the molars were evaluated at an interval of one, three, and six months.

**Figure 1:**
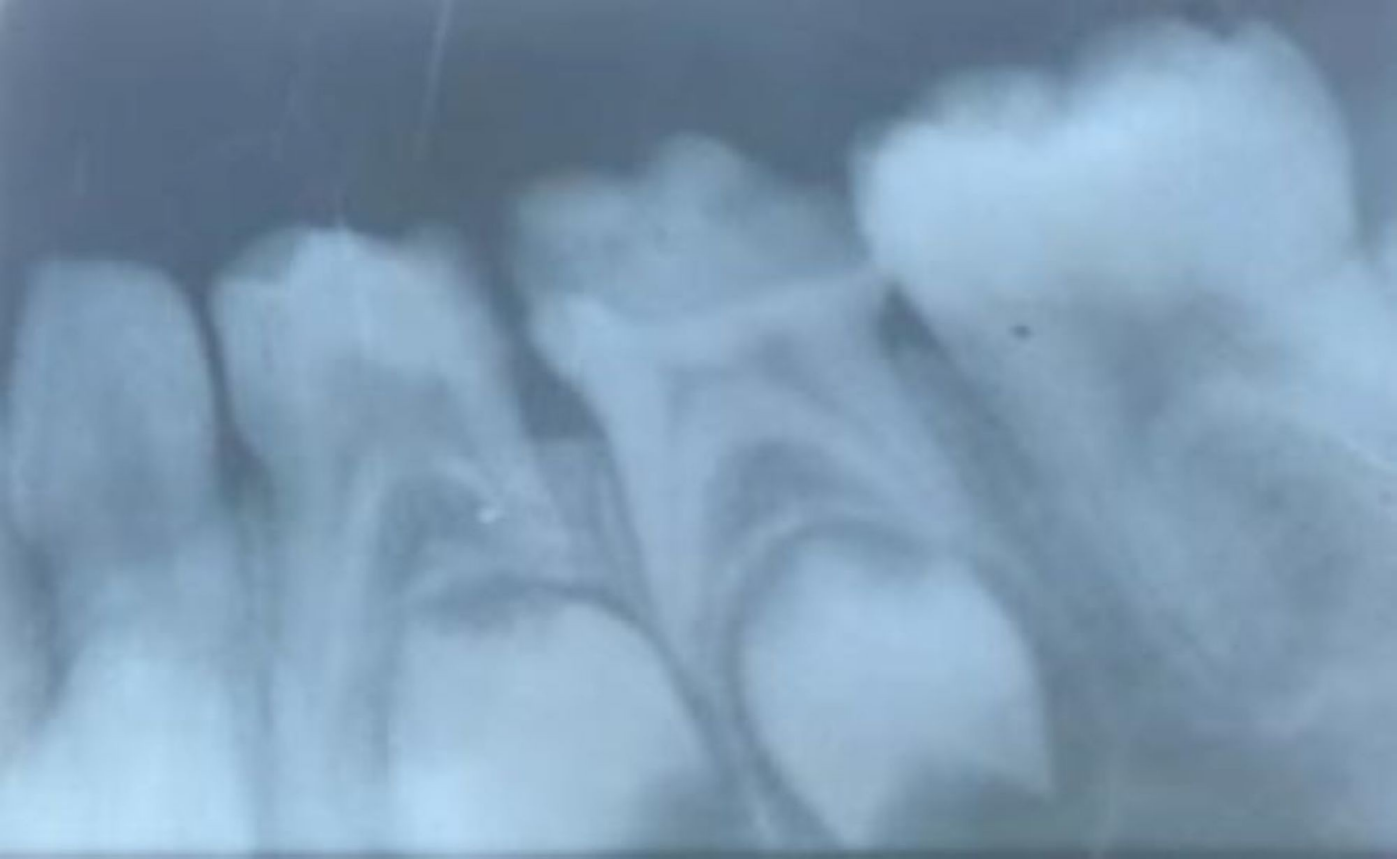
Preoperative radiograph for the placement of MTA MTA: Mineral Trioxide Aggregate

**Figure 2:**
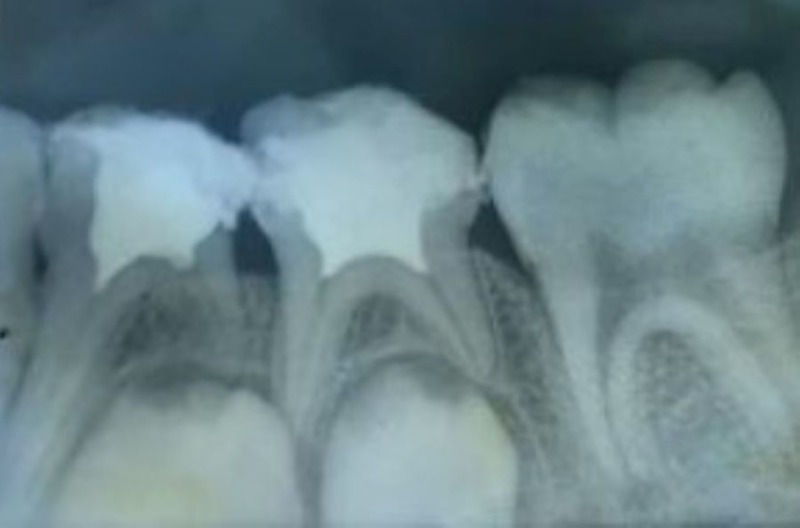
Postoperative radiograph after the placement of MTA MTA - Mineral Trioxide Aggregate

**Figure 3:**
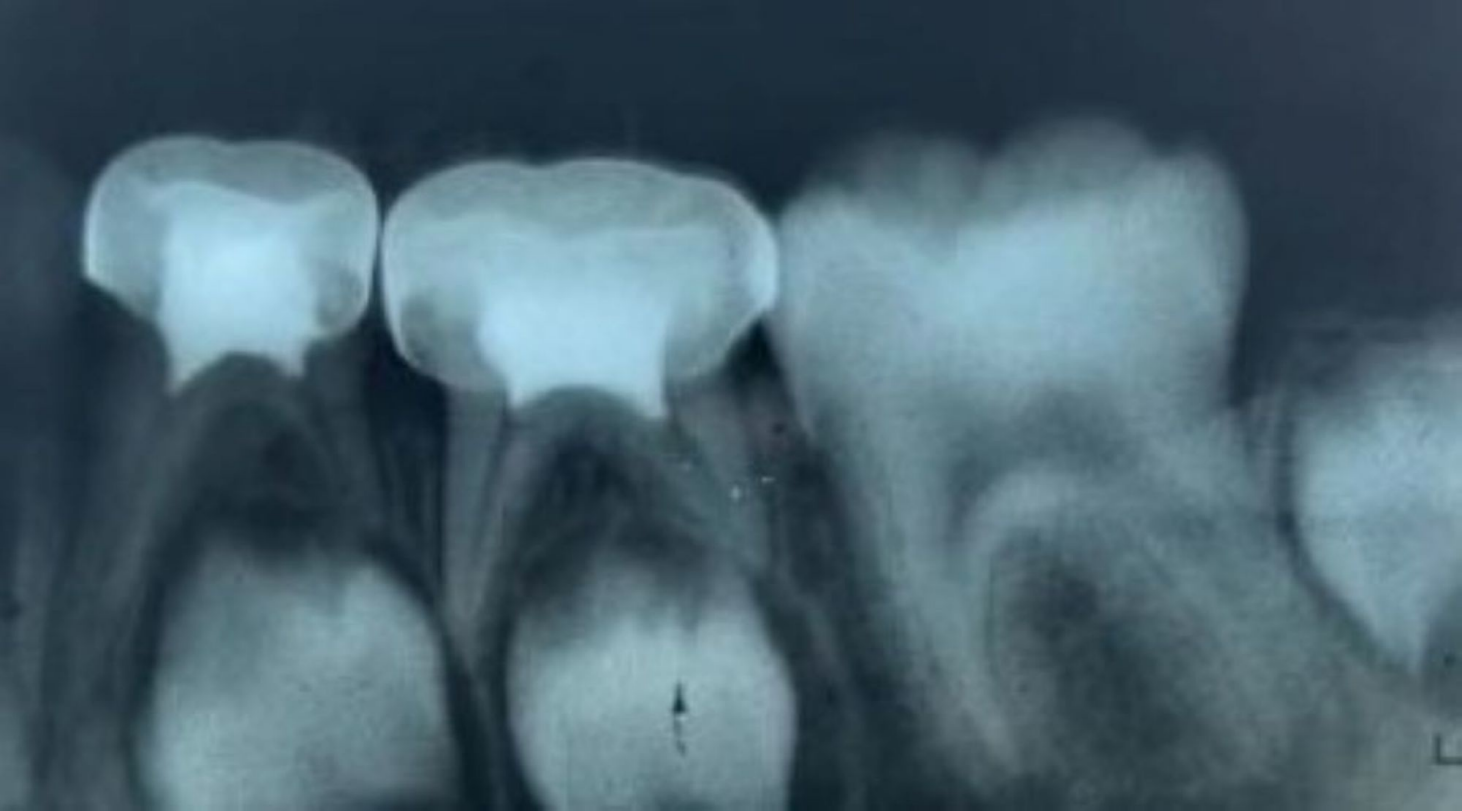
Three months follow-up after the placement of MTA MTA - Mineral Trioxide Aggregate

Figures 1-3 show the radiographs for teeth treated with MTA.

**Figure 4:**
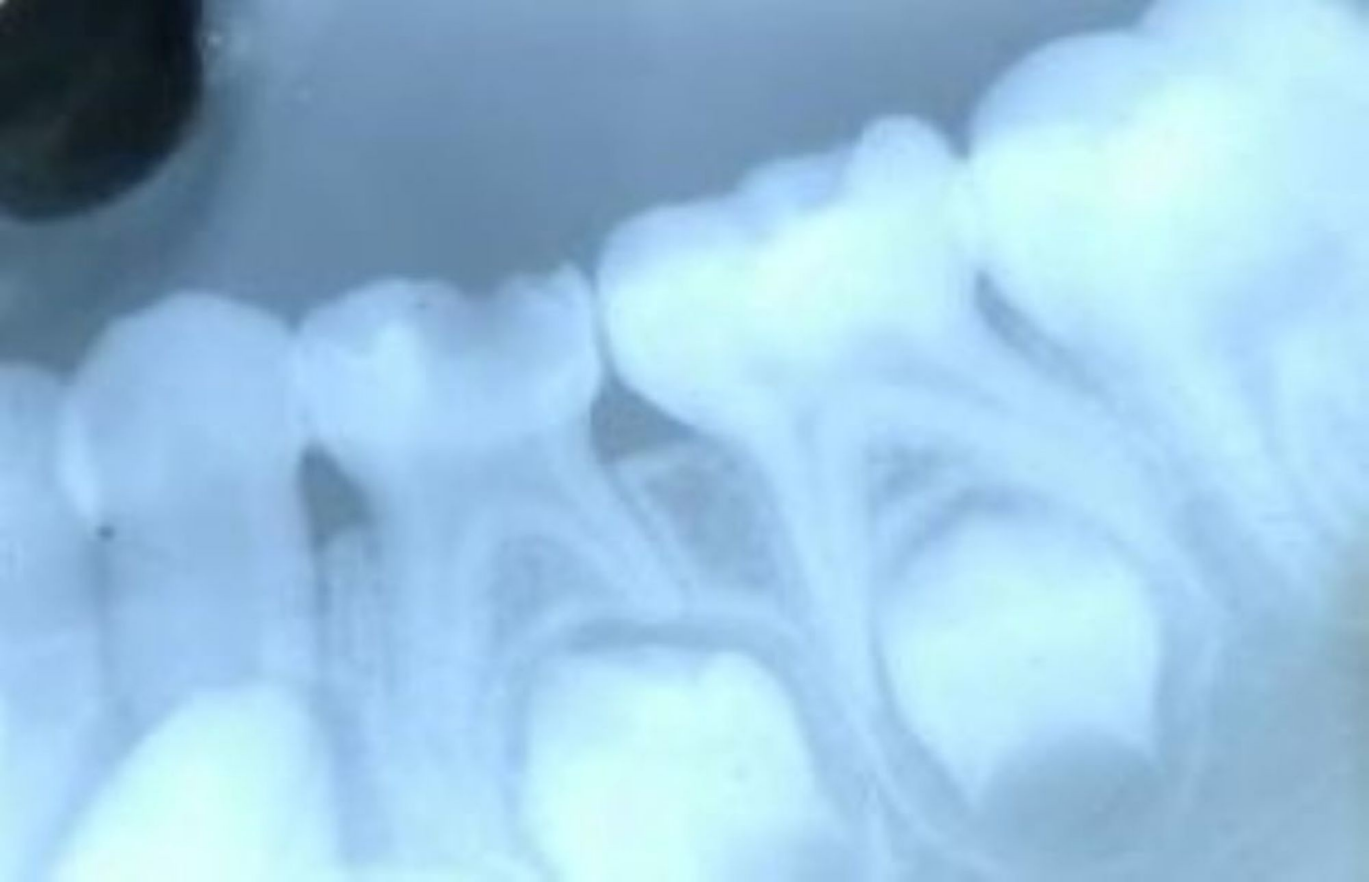
Preoperative radiograph for the placement of Biodentine

**Figure 5:**
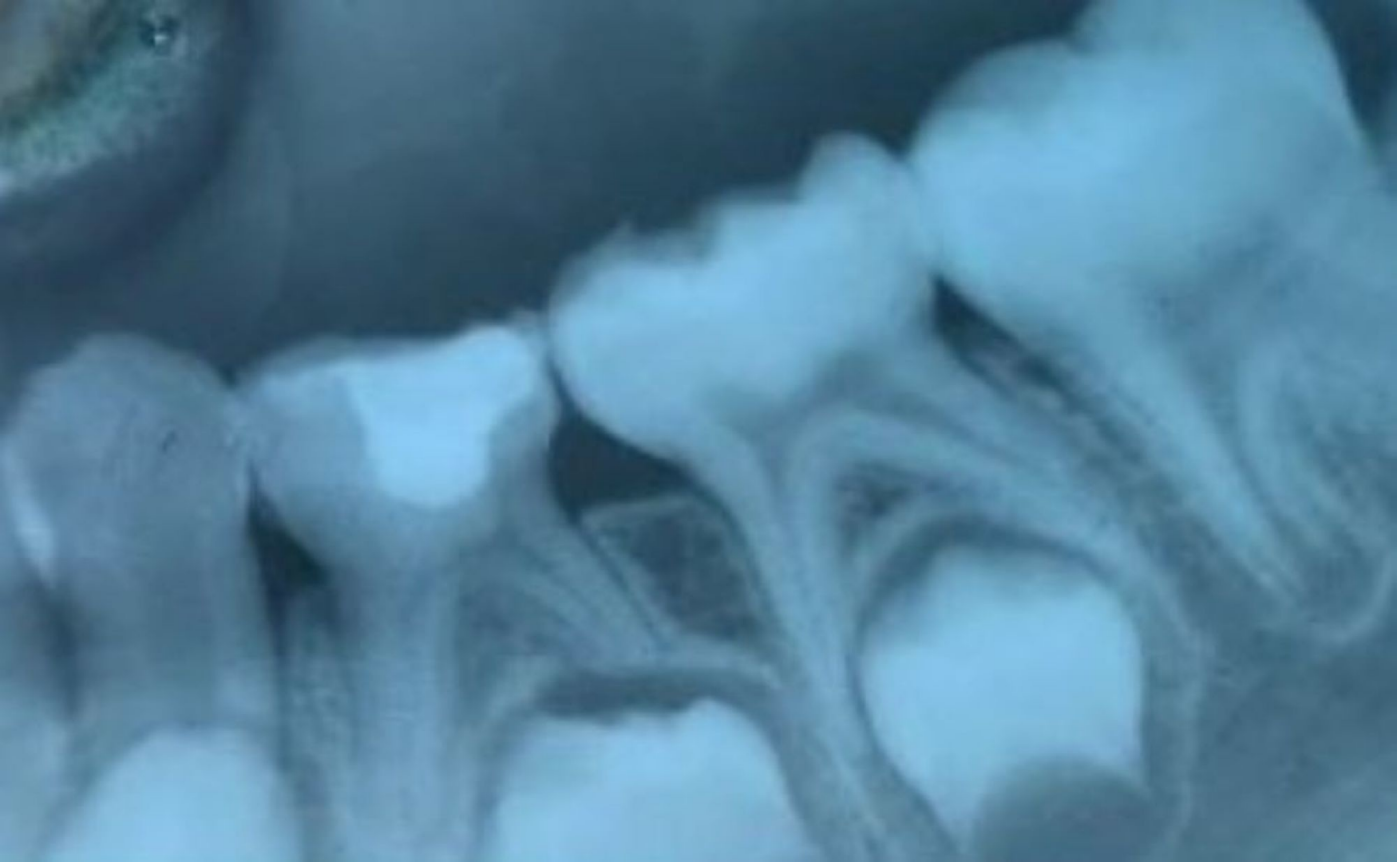
Postoperative radiograph after the placement of Biodentine

**Figure 6:**
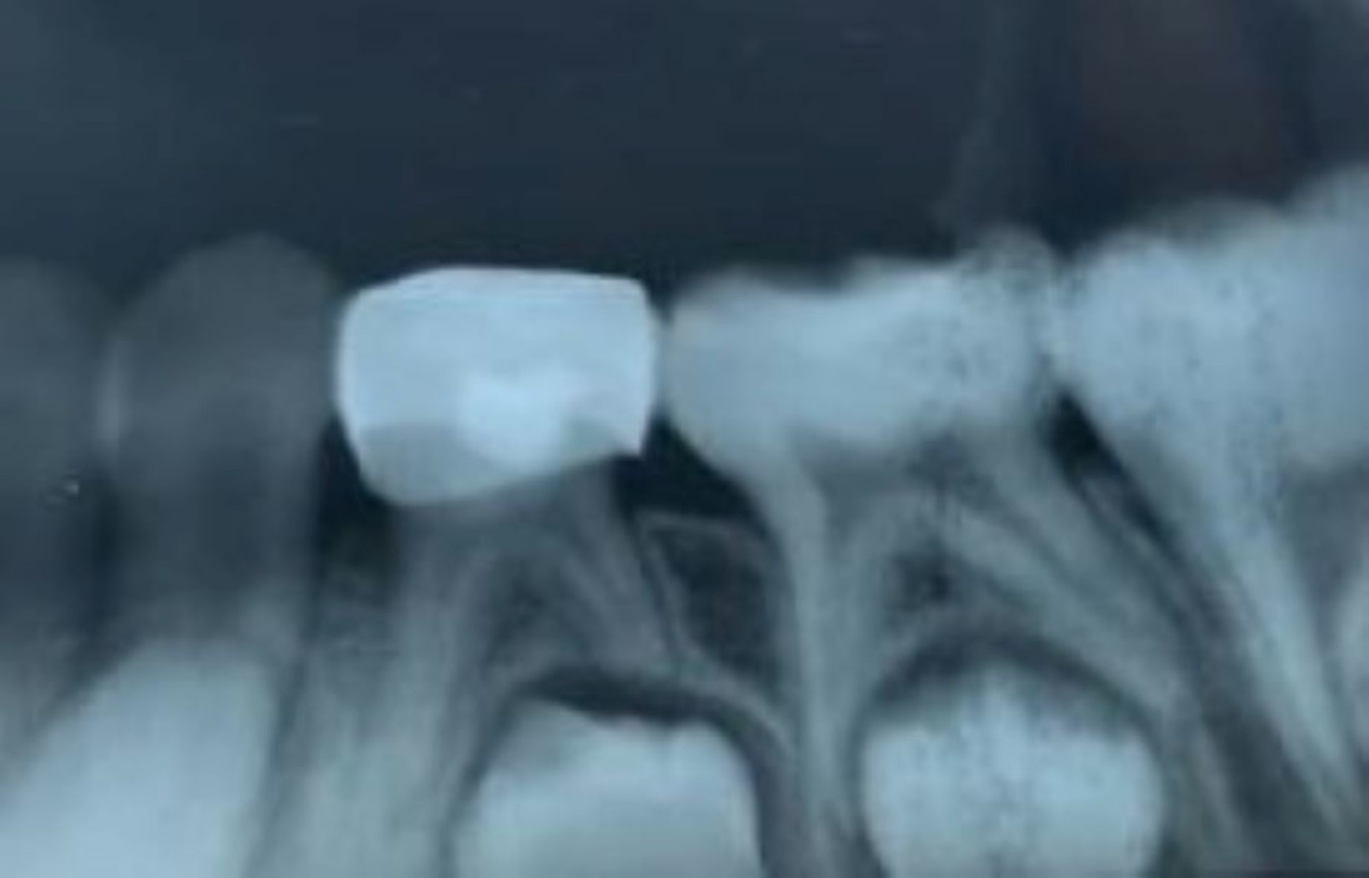
Three months follow-up after the placement of Biodentine

Figures 4-6 show the radiographs for teeth treated with Biodentine.

**Figure 7:**
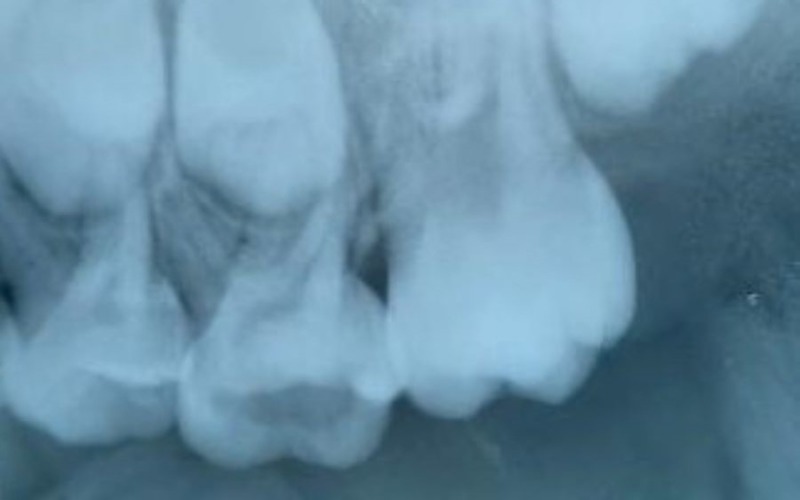
Preoperative radiograph for the placement of Pulpotec

**Figure 8:**
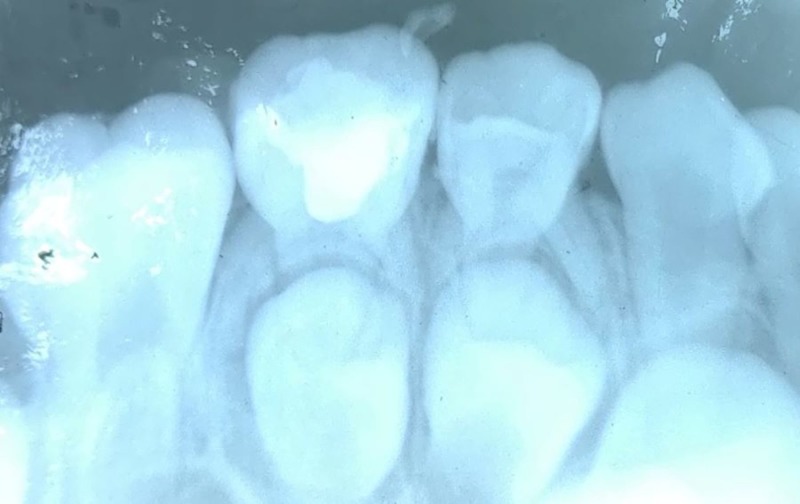
Postoperative radiograph after the placement of Pulpotec

**Figure 9:**
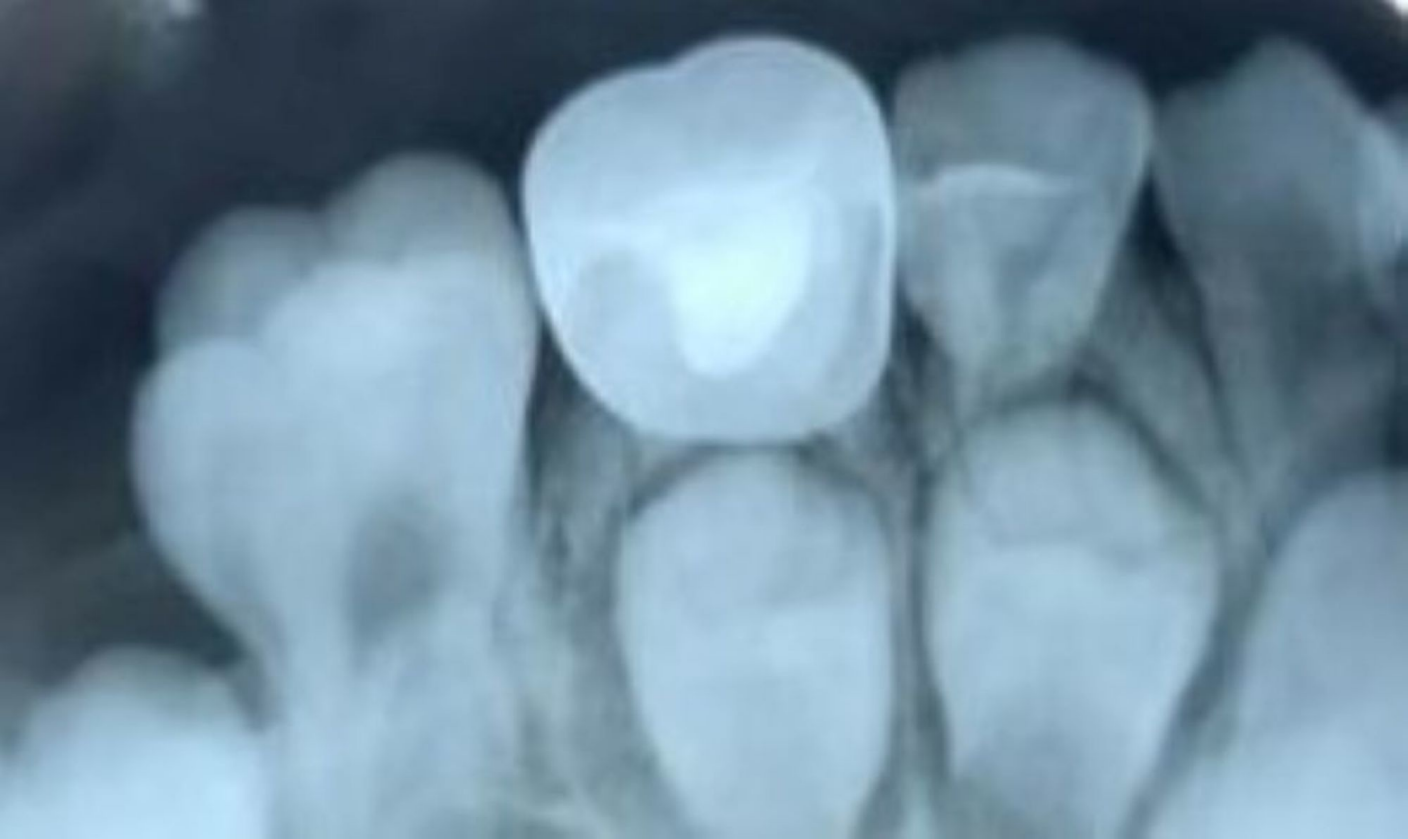
Three months follow-up after the placement of Pulpotec

Figures 7-9 show the radiographs of teeth treated with Pulpotec.

## Results

At the end of the first month, there were no negative clinical and radiographical findings observed in all three groups. By the end of one month, eight molars were lost to follow-up. After three months, 17 were lost and this number increased to 21 after six months (Table 1).

**Table 1:** Follow-up at the first, third, and sixth months MTA - Mineral Trioxide Aggregate

Follow-up	MTA	BIODENTINE	PULPOTEC
Starting	28	28	28
1^st^ month	27	25	24
3^rd^ month	22	21	24
6^th^ month	22	21	20

Among the 63 teeth that were clinically evaluated after the first, third, and sixth months, one tooth in Group I showed pain, swelling, sinus tract, and mobility by the end of three months. MTA (Group I) showed 96% clinical success by the end of three months, whereas teeth treated with Biodentine (Group II) and Pulpotec (group III) revealed no abnormal clinical findings by the end of six months. Thus, Biodentine and Pulpotec showed 100% clinical success by the end of six months (Table 2).

**Table 2:** Comparison of the clinical parameters of the three study groups AB - Absent; PT - Present; MTA - Mineral Trioxide Aggregate

Groups	Pain				Sinus tract				Swelling				Mobility				Premature exfoliation			
	3 months		6 months		3 months		6 months		3 months		6 months		3 months		6 months		3 months		6 months	
	AB	PT	AB	PT	AB	PT	AB	PT	AB	PT	AB	PT	AB	PT	AB	PT	AB	PT	AB	PT
MTA	21	1	21	1	21	1	21	1	21	1	21	1	21	1	21	1	22	0	22	0
BIODENTINE	21	0	21	0	21	0	21	0	21	0	21	0	21	0	21	0	21	0	21	0
PULPOTEC	24	0	20	0	24	0	20	0	24	0	20	0	24	0	20	0	24	0	20	0
			P=0.35				P=0.35				P=0.35				P=0.35				P=0.35	

On radiographical evaluation after the first, third, and sixth months, one tooth in Group I showed periodontal ligament widening and periapical radiolucency at the end of three months. MTA (Group I) showed 96% radiographical success by the end of three months. Among the teeth treated with Biodentine (Group II), two teeth showed periodontal ligament widening - one at the end of the third month and another at the end of the sixth month. Thus, Group II showed 90% radiographical success by the end of six months. In the case of teeth treated with Pulpotec (group III), no teeth revealed abnormal radiographical findings by the end of six months. Thus, Group III showed 100% radiographical success by the end of six months (Table 3).

**Table 3:** Comparison of the radiographical parameters of the three study groups AB - Absent; PT - Present; MTA - Mineral Trioxide Aggregate

Groups	Periodontal ligament widening				Internal/external resorption				Furcal/periapical radiolucency			
	3 months		6 months		3 months		6 months		3 months		6 months	
	AB	PT	AB	PT	AB	PT	AB	PT	AB	PT	AB	PT
MTA	21	1	21	1	22	0	22	0	21	1	21	1
BIODENTINE	20	1	19	2	21	0	21	0	21	0	21	0
PULPOTEC	24	0	23	0	24	0	23	0	24	0	23	0
			P= 0.056								P= 0.35	

A comparison of clinical criteria, i.e., status of pain, sinus tract, swelling, mobility, and premature exfoliation at the end of three and six months showed a non-significant difference (p>0.05) for each material (Tables 4-5). A comparison of radiographical criteria, i.e., periodontal ligament space widening, internal/external resorption, and furcal/periapical radiolucency at three- and six-month intervals also showed a non-significant difference (p>0.05) for each material (Tables 4-5).

**Table 4:** Postoperative evaluation after three months MTA - Mineral Trioxide Aggregate

Parameter	Group I MTA (n=22)		Group II BIODENTINE (n=21)		Group III PULPOTEC (n=24)		Significance of difference	
	n	percentage	n	percentage	n	percentage	X^2^	p
Clinical parameters								
Pain	1	4.5	0	0	0	0	2.076	0.354
Sinus tract	1	4.5	0	0	0	0	2.076	0.354
Swelling	1	4.5	0	0	0	0	2.076	0.354
Mobility	1	4.5	0	0	0	0	2.076	0.354
Premature exfoliation	0	0	0	0	0	0	-------	-------
Radiographical parameters								
Periodontal ligament widening	1	4.5	1	4.8	0	0	1.152	0.562
Internal/external resorption	0	0	0	0	0	0	-----	------
Furcal/periapical radiolucency	1	4.5	0	0	0	0	2.076	0.354

**Table 5:** Postoperative evaluation after six months

Parameter	Group I MTA (n=22)		Group II BIODENTINE (n=21)		Group III PULPOTEC (n=20)		Significance of difference	
	n	percentage	n	percentage	n	percentage	X^2^	p
Clinical parameters								
Pain	1	4.5	0	0	0	0	1.894	0.38
Sinus tract	1	4.5	0	0	0	0	1.894	0.38
swelling	1	4.5	0	0	0	0	1.894	0.38
Mobility	1	4.5	0	0	0	0	1.894	0.38
Premature exfoliation	1	4.5	0	0	0	0	1.894	0.38
Radiographical parameters								
Periodontal ligament widening	1	4.5	2	9.5	0	0	2.052	0.358
Internal/external resorption	0	0	0	0	0	0	--------	-------
Furcal/periapical radiolucency	1	4.5	0	0	0	0	1.894	0.38

## Discussion

Vital pulpotomy is considered a one-stage procedure with the objective of preserving the vitality and function of the remaining radicular portion of pulp and keeping it asymptomatic without adverse clinical signs such as sensitivity, pain, or swelling [1]. The ideal pulpotomy medicament and/or technique should be bio-inductive or at least biocompatible, bactericidal, and harmless to the pulp and surrounding structures [2].

Formocresol has been a popular pulpotomy medicament in the primary dentition and is still universally used in pulpal therapy for primary teeth. Despite its wide usage, formocresol has several disadvantages apart from not being ideal pulpotomy medicament. Concerns have been raised by several researchers over the use of formocresol in humans [3], and several alternatives have been proposed like mineral trioxide aggregate, Biodentine, and Pulpotec.

In search of ideal pulpotomy medicament, materials like mineral trioxide aggregate, Biodentine, and Pulpotec have been tried in the present study with variable clinical and radiological success.

The success rate of MTA in the present study has been promising with 96% of clinical and radiographical success in six months. The results of the study are comparable to other contemporary studies.

A similar study comparing the clinical and radiographic success rates for formocresol and MTA pulpotomy in primary molars was 83% and 97%, respectively [4]. Another study comparing the clinical and radiographical success of formocresol and MTA in which, at the end of 12 months, the clinical outcome of formocresol was 91.3% and MTA was 100%. The radiographical outcome of formocresol was 78.26% and MTA was 95.74%. Periodontal ligament widening and inter-radicular radiolucency were seen in the formocresol group. The probable reason may be due to the fixative effect of formocresol and the ability of vapors to escape via the apical foramen. Failures of MTA can be attributed to a misdiagnosis of inflammation in the radicular pulp prior to treatment [5].

The failures that are seen by the subjects treated with MTA in the present study may be due to microleakage through the restoration and failure to place a stainless steel crown.

In the present study, Group II was treated with Biodentine, which showed 100% clinical success at the six months' follow-up whereas radiographical success was 96% by the end of three months and 91% by the end of six months.

Biodentine has several advantages, which include good sealing ability, adequate compressive strength, and short setting time, which provide a significant clinical advantage over other comparable materials [6].

In a similar study comparing MTA, laser, and Biodentin for pulpotomy, the results showed failure for two cases in the laser and Biodentine groups. They explained the reason for the failures might be due to iatrogenic errors like poorly adapted stainless steel crowns, a thin base, voids in the cement, and areas of residual caries or coronal pulp tissue. It is also quite possible that the laser pulpotomy is more sensitive to operator technique. They concluded that these materials are better alternatives to formocresol [7].

While considering material for clinical use, handling characteristics are very important. Biodentine received a better rating for material handling and performance after restoration placement because of its mechanical properties and setting time as compared to MTA. Biodentin has high compressive strength, antifungal and antimicrobial activity, and is less porous compared to MTA [8].

In this study, Group III, treated with Pulpotec, showed 100% clinical and radiological success with the six months follow-up. Pulpotec is used for the rapid and long-term treatment of pulpitis by pulpotomy in vital molars, both primary and permanent [9].

A study conducted on histological evaluation following the usage of Pulpotec for pulpotomy procedure by Satygo revealed three zones next to the pulp-cement interface, which is similar to formocresol. Though Pulpotec and formocresol showed similar histological findings, due to the minimal concentration of formaldehyde in Pulpotec as compared to formocresol, Pulpotec is advisable over formocresol. Recent research on formaldehyde metabolism, pharmacokinetics, and carcinogenicity indicates that formaldehyde is probably not a potent human carcinogen under conditions of low exposure [10].

Pulpotec has the advantage of having clinical success even in cases with a little residual blood in the pulp chamber when used as a pulpotomy medicament. The results of Pulpotec are in agreement with Donskaya and Dedeyan's [11] clinical trials using Pulpotec as pulpotomy medicament and reported easiness and simplicity. The paste hardens quickly after the mixing of ingredients, providing optimal conditions for final restoration with its added hemostatic and fixative properties.

However, in the present study, Pulpotec application was simple, time-saving, and affordable and showed 100% clinical and radiographic success rate in a six-month period. This high success rates may be due to the anti-inflammatory, disinfectant, and steroidal properties of the material.

A study conducted comparing the clinical and radiographical success rates of formocresol, MTA, Pulpotec, and Emdogain showed 100% clinical and radiographical success at the end of six months. By the end of 24 months' follow-up, the clinical success rates of FC and Pulpotec were 94.4%, EMD was 83.3%, and MTA was 100% [12].

In the never-ending quest for an ideal pulpotomy agent, this study has tested three materials. Even though there was reasonable clinical and radiographical success with all the three materials, Pulpotec showed a higher success rate. Studies with long-term follow-up are needed to establish the ideal pulpotomy medicament.

## Conclusions

At the end of the study, it was observed that Group III (Pulpotec) exhibited the overall best results as the pulpotomy agent for primary molars followed by Group II (Biodentine), whereas Group I (MTA) showed the least favorable results, both clinically and radiographically. In order to get more authentic results, detailed longitudinal studies involving bigger samples and larger follow-ups are needed.
